# Increased Incidence and Plasma-Biofilm Formation Ability of SCC*mec* Type IV Methicillin-Resistant *Staphylococcus aureus* (MRSA) Isolated From Patients With Bacteremia

**DOI:** 10.3389/fcimb.2021.602833

**Published:** 2021-03-26

**Authors:** Masakaze Hamada, Tetsuo Yamaguchi, Ayami Sato, Daisuke Ono, Kotaro Aoki, Chiaki Kajiwara, Soichiro Kimura, Tadashi Maeda, Masakazu Sasaki, Hinako Murakami, Yoshikazu Ishii, Kazuhiro Tateda

**Affiliations:** ^1^Department of Microbiology and Infectious Diseases, Toho University School of Medicine, Tokyo, Japan; ^2^Department of Surgery, Toho University Sakura Medical Center, Chiba, Japan; ^3^Department of Infectious Diseases and Infection Control, Saitama Medical Center, Saitama Medical University, Saitama, Japan; ^4^Department of General Medicine and Emergency Care, Toho University Omori Medical Center, Tokyo, Japan; ^5^Department of Clinical Laboratories, Toho University Omori Medical Center, Tokyo, Japan

**Keywords:** *Staphylococcus aureus*, Staphylococcal cassette chromosome *mec* (SCC*mec)* type IV, methicillin-resistant *Staphylococcus aureus* (MRSA), biofilm, plasma, bloodstream infection

## Abstract

In Japan, Staphylococcal cassette chromosome *mec* (SCC*mec*) type IV methicillin-resistant *Staphylococcus aureus* (MRSA) is an increasingly prominent cause of bacteremia, but the virulence of most of these strains is unclear. We aimed to investigate the relationship between the molecular characteristics and the ability to form biofilms in the presence of blood plasma (plasma-biofilms) of MRSA strains isolated from bloodstream infections. In this study, the molecular characteristics and biofilms of MRSA strains isolated from blood cultures between 2015 and 2017 were analyzed by PCR-based assays, crystal violet staining, and confocal reflection microscopy methods. Among the 90 MRSA isolates, the detection rate of SCC*mec* type II clones decreased from 60.7 to 20.6%. The SCC*mec* type IV clone replaced the SCC*mec* type II clone as the dominant clone, with a detection rate increasing from 32.1 to 73.5%. The plasma-biofilm formation ability of the SCC*mec* type IV clone was higher than the SCC*mec* type II clone and even higher in strains harboring the *cna* or *arcA* genes. Plasma-biofilms, mainly composed of proteins, were formed quickly and strongly. Our study demonstrated the increased plasma-biofilm formation ability of SCC*mec* type IV strains.

## Introduction

Methicillin-resistant *Staphylococcus aureus* (MRSA) was first reported in 1961 (Jevons, 1961). Over the subsequent decades, hospital-associated MRSA (HA-MRSA) has spread, remaining of great concern as a cause of infection in immunocompromised hospital patients, affecting hospitals and healthcare facilities worldwide. In the 1990s, community-associated MRSA (CA-MRSA) emerged as another apparent threat (Udo et al., 1993). CA-MRSA exhibits hyper-virulence and has been reported to circulate in communities through the infection and/or colonization of healthy individuals, particularly children and young adults.

Outbreaks of CA-MRSA have been reported worldwide (DeLeo et al., 2010); especially prevalent in the United States, and approximately 80% of *S. aureus* isolates from skin and soft tissue infections (SSTIs) are MRSA (Miller et al., 2005). Most of them are of a single CA-MRSA genotype called the USA300 clone (ST8/Staphylococcal cassette chromosome *mec* (SCC*mec*) type IV), which causes not only SSTIs but also lethal infections such as necrotizing pneumonia and sepsis (Nimmo, 2012; Prosperi et al., 2013). Furthermore, the USA300 clone that has spread throughout community settings also invades hospital-associated settings and has become as prominent a pathogen as HA-MRSA, causing nosocomial infections.

Japan is an HA-MRSA-endemic country; however, the methicillin resistance ratio of *S. aureus* has dropped from nearly 70% in 2000 to less than 50% today (Japan Nosocomial Infections Surveillance^1^). We previously clarified that the methicillin resistance ratio of *S. aureus* isolates from SSTIs in communities is approximately 20%, which is significantly lower than in the United States (Yamaguchi et al., 2015). Furthermore, most of the CA-MRSA strains are SCC*mec* type IV but are neither USA300 clones nor Panton-Valentine Leucocidin (PVL)-producing strains. The virulence of other SCC*mec* type IV clones, except specific clones such as the USA300 clone, is not clear.^1^

Among the cases in which *S. aureus* has been isolated from blood culture samples at the Toho University Omori Medical Center in Tokyo, Japan, the methicillin resistance rate has dropped from 66.7% in 2007 to 33.3% in 2013. However, the methicillin resistance rate has re-risen since 2015, reaching 45.9% in 2017. The same trend has also been observed in other institutions in Japan (Miura et al., 2018), thus investigating what is causing the increase in the number of MRSA cases is crucial.

The biofilm formed by bacteria is involved in establishing bacteremia, including device-related infections (Hoiby et al., 2015), and that bacterial biofilms comprise a variety of components and substances from both the bacteria and the host (Alhede et al., 2014; Zapotoczna et al., 2016). Especially, *S. aureus* possesses a specific virulence factor called coagulase, which is thought to play a significant role in biofilm formation in *S. aureus* bloodstream infections. Coagulase binds to host prothrombin and forms active staphylothrombin complexes, which convert soluble monomeric fibrinogen into self-polymerizing insoluble fibrin and activate a coagulation cascade.

The plasma-biofilm is a recently proposed concept and refers to the biofilm formed in the presence of plasma (Besser et al., 2019). In recent years, some reports have suggested that *S. aureus* uses the fibrin and fibrinogen recruited by coagulase to form the biofilm scaffold in the host’s blood (Gronnemose et al., 2017; Besser et al., 2019). We noticed differences in the plasma-biofilm formation ability between different clones in our previous study (Sato et al., 2019). It is assumed that if the ability of each strain to form plasma-biofilm differs *in vivo*, MRSA strains with a high level of plasma-biofilm formation ability would exhibit high virulence in the blood.

In this study, we investigated the molecular characteristics of MRSA strains isolated from blood culture samples between 2015 and 2017, a period during which MRSA isolation was on the increase in our medical facility. To assess the pathogenicity of MRSA in the bloodstream, the biofilms of each MRSA strain formed in the presence of plasma were compared by the crystal violet (CV) staining method (Merritt et al., 2005) and confocal reflection microscopy (CRM) method (Yawata et al., 2010). This study aimed to clarify the relationship between the molecular characteristics and plasma-biofilm formation ability of MRSA strains isolated from bacteremia cases.

## Materials and Methods

### Bacterial Strains

Between January 2007 and December 2017, *S. aureus* strains were isolated from blood culture samples from 796 cases at the Clinical Microbiology Laboratory at the Toho University Omori Medical Center in Tokyo, Japan. If multiple *S. aureus* isolates were present in various samples from the same patient, only one isolate was selected; however, if isolates from the same patient showed different antimicrobial susceptibility, each was treated as a separate case.

Between 2015 and 2017, MRSA strains were isolated from blood cultures from 98 cases (33 in 2015, 31 in 2016, and 34 in 2017), and genetic analyses such as SCC*mec* typing and virulence gene detection were performed on 90 MRSA strains (28 in 2015, 28 in 2016, and 34 in 2017) that had been stored. Two strains of MRSA, ATCC BAA-1556 (FPR3757 strain; USA300 clone) and N315 (New York/Japan clone), were positive controls for virulence gene detection and biofilm formation analysis. This study was approved by the Ethics Committee of the Faculty of Medicine, Toho University (approval number A17114), and pathogen protocols were approved by the Toho University Safety Committee for Pathogens (approval number 18-43-87).

### Molecular Characterization

Each isolate was cultured overnight in brain heart infusion (BHI) broth (Eiken Chemical Co., Tokyo, Japan), and the cells were harvested by centrifugation at 8,000×*g* for 1 min. Bacterial genomic DNA was then extracted using a DNeasy Blood & Tissue Kit (QIAGEN, Valencia, CA, USA) with lysostaphin (Wako, Osaka, Japan). Genomic DNA was a template for PCR-based screening assays. MRSA SCC*mec* types were determined using a PCR-based assay as previously described (Kondo et al., 2007; Yamaguchi et al., 2020). The two multiplex PCR sets identify the *mec* gene complex type and *ccr* gene complex type, and the SCC*mec* type is determined by the combination pattern of the *mec* gene complex type and *ccr* gene complex type. The SCC*mec* type is classified from I to XIV; however, this method can only be used to determine type I to VI. Because this method could not be used to determine the other SCC*mec* types, including types VII–XIV, isolates for which the SCC*mec* type could not be identified were treated as non-typeable (NT), meaning these isolates were SCC*mec* types VII–XIV, or new types.

Staphylococcal virulence genes were also detected using a PCR assay, using reported primers (Jarraud et al., 2002; Diep et al., 2006). The target virulence genes included the PVL-encoding gene (*lukSF-PV*), the toxic shock syndrome toxin-1 (TSST-1) gene (*tst*), and the arginine catabolic mobile element (ACME) gene (*arcA*). PCR assays to identify microbial surface components recognizing adhesive matrix molecule (MSCRAMM) genes were also performed, following described protocols (Tristan et al., 2003; Vancraeynest et al., 2004). The MSCRAMM-encoding genes included *fnbA*, *fnbB*, *fib*, *clfA*, *clfB*, *bbp*, *cna*, *eno*, and *ebps*.

### Quantification of Biofilms by CV Staining Assay

For routine cultures, 27 MRSA strains from 2016, BAA-1556 or N315, were grown oxically overnight on BHI agar (Becton Dickinson, Franklin Lakes, NJ, USA) at 35°C. After incubation, the colonies were inoculated in BHI broth and then grown oxically for 12 h at 35°C with shaking at 160 rpm. Shaking cultures were diluted to 1:500 with tryptic soy broth (Becton Dickinson, Franklin Lakes, NJ, USA) containing 0.5% (w/v) glucose (TSBG), and the diluted pre-cultures were diluted 1:1 with TSBG or TSBG containing 14.28% (v/v) “Eiken” rabbit plasma (Eiken Chemical Co., Tokyo, Japan). The final plasma concentrations were 7.14%. Next, 100 μl aliquots were inoculated in 96-well round-bottom polystyrene plates and statically incubated at 35°C for 1, 2, 6, or 24 h under aerobic conditions. MRSA biofilms formed in 96-well plates were quantified following a reported CV staining method (Merritt et al., 2005); the biofilms were washed with phosphate-buffered saline (PBS) and stained with 150 µl of 0.2% (w/v) CV solution. CV-stained biofilms were washed with PBS and solubilized with 150 µl of 33.3% (v/v) acetic acid. The eluents were transferred to fresh 96-well plates to determine the absorbance at 595 nm (*A*_595_). Sometimes, the eluents were diluted to 1:10 with acetic acid.

In the biofilm formation tests in the presence of extracellular matrix (ECM)-degrading agents, 100 μg/ml DNase I (Roche Diagnostics, Mannheim, Germany) or proteinase K (Sigma Aldrich, St. Louis, MO, USA) were added to TSBG or TSBG-containing plasma. After incubation for 6 h in the presence of these ECM-degrading agents, biofilm formation was measured using the CV staining method. DNase I was solubilized in 150 mM NaCl and 1 mM CaCl_2_; proteinase K was solubilized in 20 mM Tris-HCl buffer (pH 7.5) and 100 mM NaCl (Gutierrez et al., 2014).

### Visualization of Biofilms by CRM

MRSA shaking cultures were diluted to 1:500 with TSBG, and the diluted pre-cultures were diluted to 1:1 with TSBG-containing rabbit plasma. Then, 300 μl aliquots were inoculated in 8-well coverglass chambers (IWAKI, Tokyo, Japan). After incubation at 35°C for 6 h under aerobic conditions, biofilms that formed at the bottom of glass chambers were gently washed with PBS, and the 3D structures were visualized with a CRM method (Yawata et al., 2010). A Carl Zeiss Laser Scanning Microscope (LSM 710) equipped with a 63×/1.40 numerical aperture Plan-Apochromat objective (Carl Zeiss Microscopy GmbH, Jena, Germany) was used to acquire CRM images. Biofilms were illuminated with a 514 nm argon laser, and the reflected light was collected through a 505–530 nm band-pass filter. An NT 80/20 half mirror was a beam splitter. The biomass calculation of biofilm structures was performed using a COMSTAT2 program^2^ (Heydorn et al., 2000).

### Measurement of Biofilm-Related Gene Transcription by Reverse-Transcription Quantitative PCR (RT-qPCR)

*fnbB*, *arcA*, *cna*, and *bbp* were found to be strain-specific virulence factors, and the strains that possessed these genes exhibited higher ability of plasma-biofilm formation. Next, we compared the transcript levels of the nonspecific factor genes, because differences in protein production might influence the biofilm formation. Target genes included the regulatory genes (*sarA, agrA, rnaIII, saeR*, and *sigB*), four MSCRAMM-encoding genes such as fibronectin-binding protein (*fnbA*) and fibrinogen-binding protein (*fib, clfA*, and *clfB*), and coagulase (*coa*) and staphylokinase (*sak*) genes involved in the synthesis and degradation of fibrin. These genes were present in all strains. We had already confirmed the presence of the genes *fnbB*, *arcA*, *cna*, and *bbp*, and we knew that there were strains that did not possess the genes. Even if the transcript levels of these genes were evaluated, it was clear that the strains that did not carry the genes would not respond to PCR. Therefore, we excluded the strain-specific virulence factors (*fnbB*, *arcA*, *cna*, and *bbp*) from the list of target genes evaluated for transcript levels using RT-qPCR. MRSA shaking cultures were diluted to 1:500 with TSBG, and the diluted pre-cultures were diluted to 1:1 with TSBG or TSBG-containing rabbit plasma. One milliliter aliquots were then inoculated on 12-well flat-bottom polystyrene plates. After incubation at 35°C for 1 h under aerobic conditions, 2 ml of RNAprotect (QIAGEN, Valencia, CA, USA) were added to 1 ml of static cultures, and then biofilms that formed at the bottom of the 12-well plates were scraped by vigorous pipetting. Bacterial suspensions including both non-adherent cells and biofilm cells were collected into conical tubes. To ensure a sufficient RNA yield, samples were collected from static cultures in two wells.

Bacterial pellets after centrifugation were kept at -80°C. The pellets were suspended in 0.1 ml of Tris-EDTA buffer supplemented with 0.2 mg/ml of lysostaphin and then incubated at 37°C for 30 min. Extraction of bacterial RNA was performed using the RNeasy Mini Kit (QIAGEN, Valencia, CA, USA), following the manufacturer’s protocol. After extraction, RNA samples were treated with TURBO DNase (Ambion^®^, Thermo Fisher Scientific, MA, USA), and RNA concentrations in the samples were confirmed using BioSpec-nano (Shimadzu, Tokyo, Japan). cDNA was acquired using the High Capacity cDNA Reverse Transcription Kit (Applied Biosystems™, Thermo Fisher Scientific, MA, USA) and mixed with a Fast SYBR Green Master Mix (Applied Biosystems™, Thermo Fisher Scientific, MA, USA). Finally, RT-qPCR was performed to quantify the transcription of biofilm-related genes using the Applied Biosystems 7500 Fast Real-Time PCR System. Reported oligonucleotide primers were used for RT-qPCR (Sabet et al., 2006; Burian et al., 2010; Kaito et al., 2011; Atshan et al., 2013; Ferreira et al., 2013; Schilcher et al., 2016; Tranchemontagne et al., 2016). PCR conditions were 95°C for 20 s, 40 cycles at 95°C for 3 s and 60°C for 30 s. Ct values were calculated using the 7500 Fast software version 2.3 (Applied Biosystems™, Thermo Fisher Scientific, MA, USA). The data were analyzed using the ΔΔCt method. The 16S rRNA gene was an internal standard.

### Statistical Analysis

The experiment was repeated at least twice under the same conditions. One-way ANOVA followed by Tukey’s multiple comparison test was performed using GraphPad Prism (version 6.0h, GraphPad Software Inc., San Diego, CA, USA). *P <*0.05 were considered statistically significant.

## Results

### Molecular Characterization

**Figure 1:**
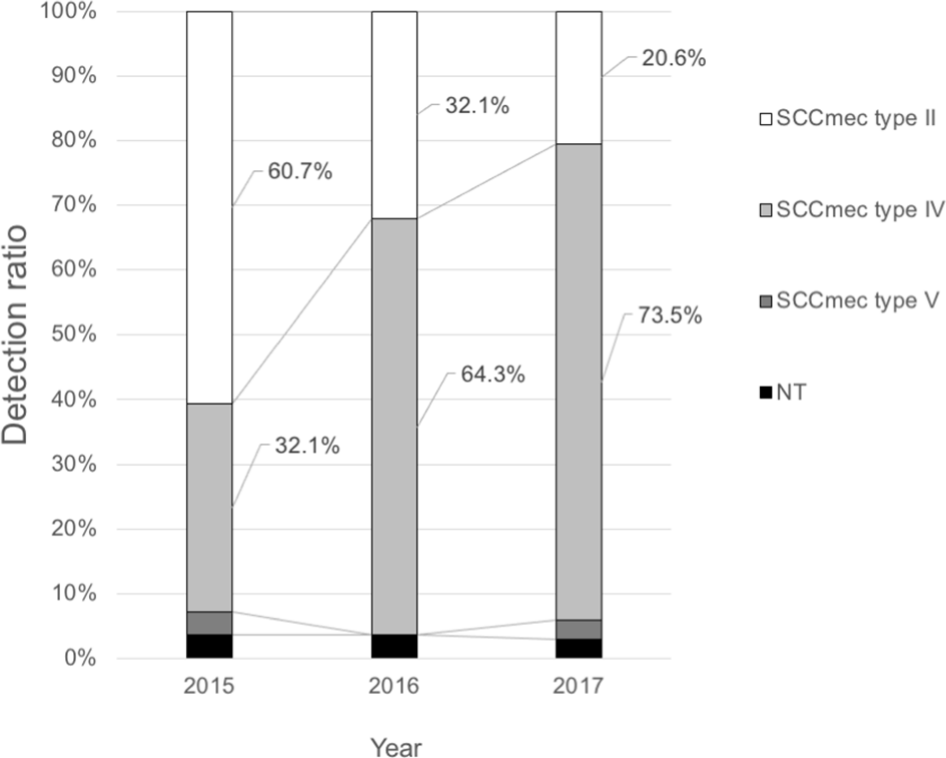
Trends of the SCC*mec* types of methicillin-resistant *Staphylococcus aureus* from blood culture samples at the Toho University Omori Medical Center.

SCC*mec* typing of 90 clinical MRSA isolates from the blood by year showed that the detection rates of SCC*mec* type II isolates classified as the HA-MRSA genotype decreased dramatically from 60.7% in 2015 to 20.6% in 2017 (**Figure 1**). SCC*mec* type IV isolates classified as the CA-MRSA genotype replaced SCC*mec* type II isolates as the dominant clone (73.5% in 2017). Of the virulence genes among SCC*mec* type IV strains between 2015 and 2017, *lukSF-PV* was only detected in four strains (4.4%), whereas *tst* was identified in 12 strains (13.3%).

The SCC*mec* type, virulence gene, and adherence gene combination was used to classify 27 isolates from 2016 into five clone types (**Table 1**). All SCC*mec* type II strains were positive for the *fnbA*, *fib*, *clfA*, *clfB*, *eno*, and *ebps* genes and negative for the *fnbB*, *bbp*, and *cna* genes. SCC*mec* type II strains were classified as clone type A or B whether they possessed the *arcA* gene. Strains classified as clone type A included those that were SCC*mec* type II and *arcA*-negative characteristics shared by the N315 clone. Strains classified as clone type B included those that were SCC*mec* type II and *arcA*-positive.

**Table 1:** Molecular characteristics of methicillin-resistant *Staphylococcus aureus* (MRSA) strains used in this study (isolated in 2016).

SCC*mec* type	Clone type	Number of strains	Adherence gene					Virulence gene	
			*arcA*	*cna*	*fnbB*	*fnbA*, *fib*, *clfA*, *clfB*, *eno*, and *ebpS*	*bbp*	*luk_SF_-PV*	*tst*
II	N315*^a^*	Reference	–	–	–	+	–	–	+
	A	7	–	–	–	+	–	–	+/-
	B	2	+	–	–	+	–	–	–
IV	C	8	–	–	+	+	–	–	+/-
	D	7	–	+	–	+	–	–	–
	E	3	+	–	+	+	–	+	–
	BAA-1556*^b^*	Reference	+	–	+	+	–	+	–

a This strain is the New York/Japan clone known as hospital-associated MRSA and is used as a reference strain of SCCmec type II.

b This strain is the USA300 clone known as community-associated MRSA and is used as a reference strain of SCCmec type IV.

All SCC*mec* type IV strains were positive for the *fnbA*, *fib*, *clfA*, *clfB*, *eno*, and *ebps* genes but negative for the *bbp* gene. SCC*mec* type IV strains were classified into three types according to the presence of the *fnbB*, *cna*, and *arcA* genes. Eight isolates identified as clone type C (SCC*mec* type IV, *arcA*-negative, *cna*-negative, and *fnbB*-positive isolates) were the most frequently detected. Clone type D strains included SCC*mec* type IV, *arcA*-negative, *cna*-positive, and *fnbB*-negative isolates. Clone type E strains included SCC*mec* type IV, *arcA-*positive, *cna*-negative, and *fnbB*-positive isolates, characteristics shared by the USA300 clone.

### Biofilm Formation

**Figure 2:**
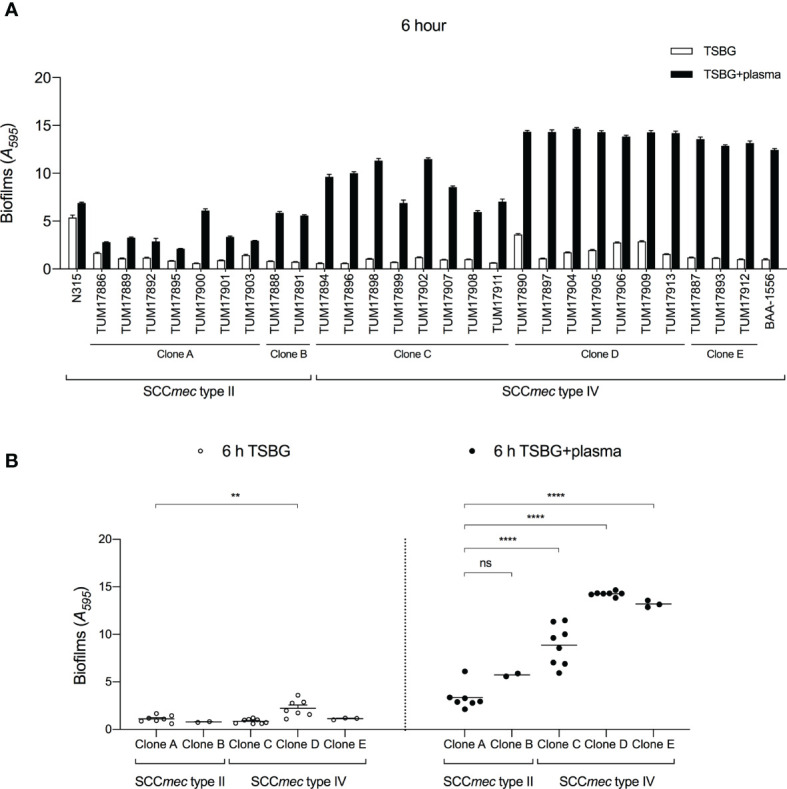
Quantification of biofilms by crystal violet (CV) staining assay. **(A)** Amount of biofilm formation of each strain with or without plasma. **(B)** Comparison of biofilm formation between clones. Three wells were used for each strain per experiment, and the experiment was conducted three times. Data from the total of nine experiments were compared between strains. **p < 0.005, ****p < 0.0001; one-way ANOVA followed by Tukey’s multiple comparison test was used in **(B)**. TSBG, tryptic soy broth containing 0.5% glucose; ns, not significant.

Molecular characteristic analysis suggested that the SCC*mec* type IV clone has been the dominant clone causing bacteremia. A previous report showed that *S. aureus* forms mature biofilms in the presence of blood plasma (Sato et al., 2019), so we compared the biofilm formation of 18 SCC*mec* type IV isolates in plasma with that of 9 SCC*mec* type II isolates after 6 h. **Figure 2A** shows the biofilm formation of each isolate, and **Figure 2B** shows the distribution of biofilm formation of clone types A–E. In the absence of plasma, the biofilm formation of SCC*mec* type IV clones was like that of SC*Cmec* type II clones. In the presence of plasma, the biofilm of each isolate was thicker than in the absence of plasma, and SCC*mec* type IV clones formed more mature biofilms than SCC*mec* type II clones (**Figures 2A, B****)**. Similar results were also observed after 24 h when biofilm formation in the absence of plasma reached the near-maximum level (**Supplementary Figures 1A, B**).

Clone type D (SCC*mec* type IV, *cna*-positive) showed the highest level of biofilm formation ability among the five clones, regardless of the presence or absence of plasma. Clone type E (SCC*mec* type IV, *arcA*/*fnbB*-positive) showed a relatively high level of biofilm formation ability in the presence of plasma (**Figure 2B**). Clone type C (SCC*mec* type IV, *fnbB*-positive) also formed more mature biofilms than clone types A and B (SCC*mec* type II) in the presence of plasma. These results indicate that the *cna*, *arcA*, and *fnbB* genes are involved in the biofilm formation ability of SCC*mec* type IV clones in plasma.

**Figure 3:**
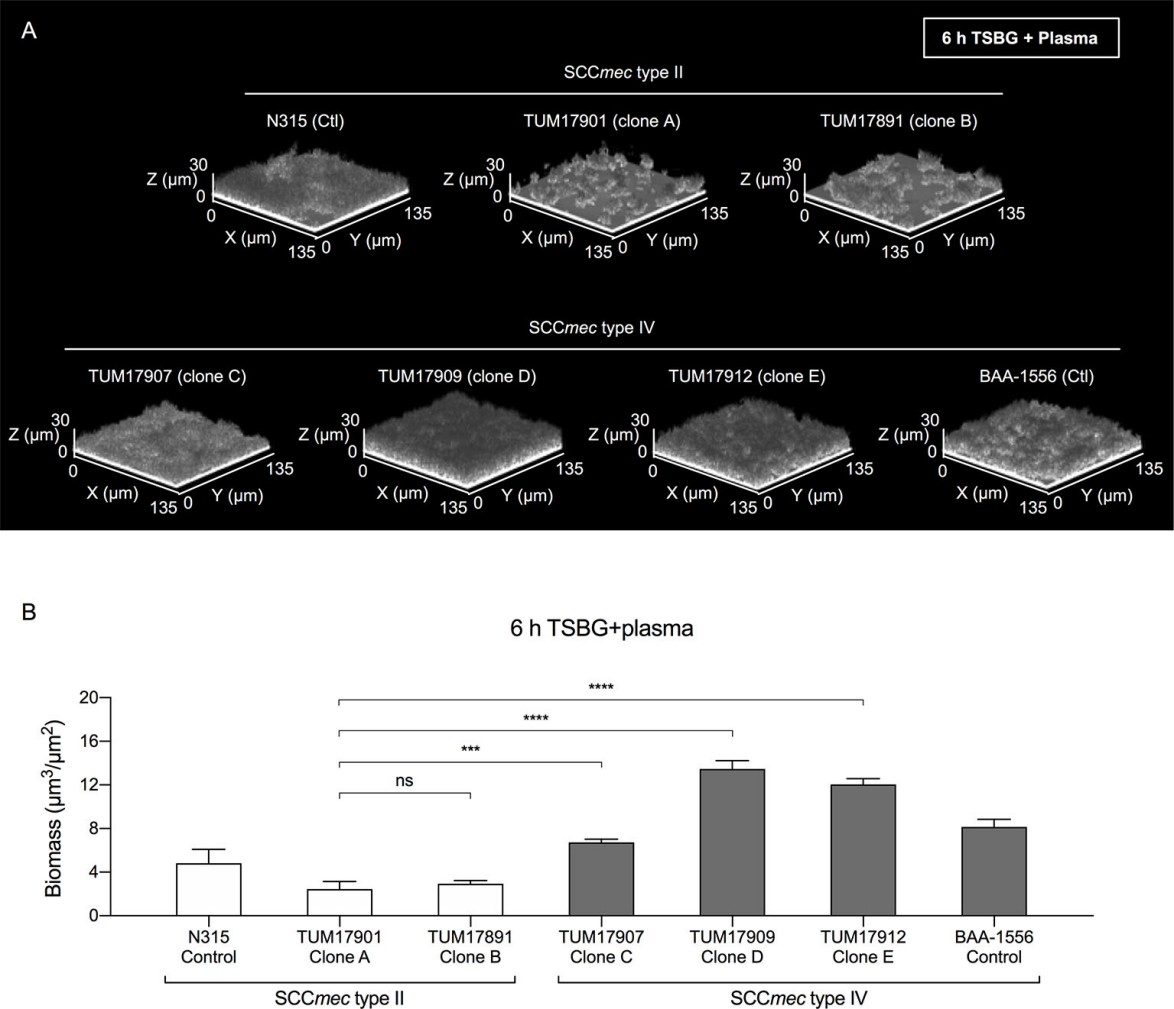
Visualization of biofilms by confocal reflection microscopy (CRM). **(A)** Biofilm in the presence of plasma (Plasma-biofilm) structures of the strain selected from each clone. **(B)** Comparison of the biomass of plasma-biofilms calculated by image analysis. Using a CRM method, the plasma-biofilm structures of each experimental strain and control strain (BAA-1556 and N315) were observed, and the biomass was measured by image analysis using a COMSTAT2 program. The biomass of plasma-biofilms was calculated in four fields of view and the means were compared. The results were confirmed by two independent experiments. ***p < 0.001, ****p < 0.0001; one-way ANOVA followed by Tukey’s multiple comparison test was used in **(B)**. TSBG, tryptic soy broth containing 0.5% glucose; ns, not significant. Fields: 135×135×30 μm (*xyz*) are indicated.

To investigate the details of the biofilm formation mechanism in plasma, one representative strain that formed an average biofilm was selected from each clone (clone A, TUM17901; clone B, TUM17891; clone C, TUM17907; clone D, TUM17909; and clone E, TUM17912). Similar to the CV staining assay results (**Figure 2A**), TUM17907 (clone C), TUM17909 (clone D), and TUM17912 (clone E) formed more mature mat-like biofilm structures than SCC*mec* type II strains including TUM17901 (clone A) and TUM17891 (clone B) (**Figures 3A, B**).

### Biofilm Formation in the Presence of ECM-Degrading Agents

**Figure 4:**
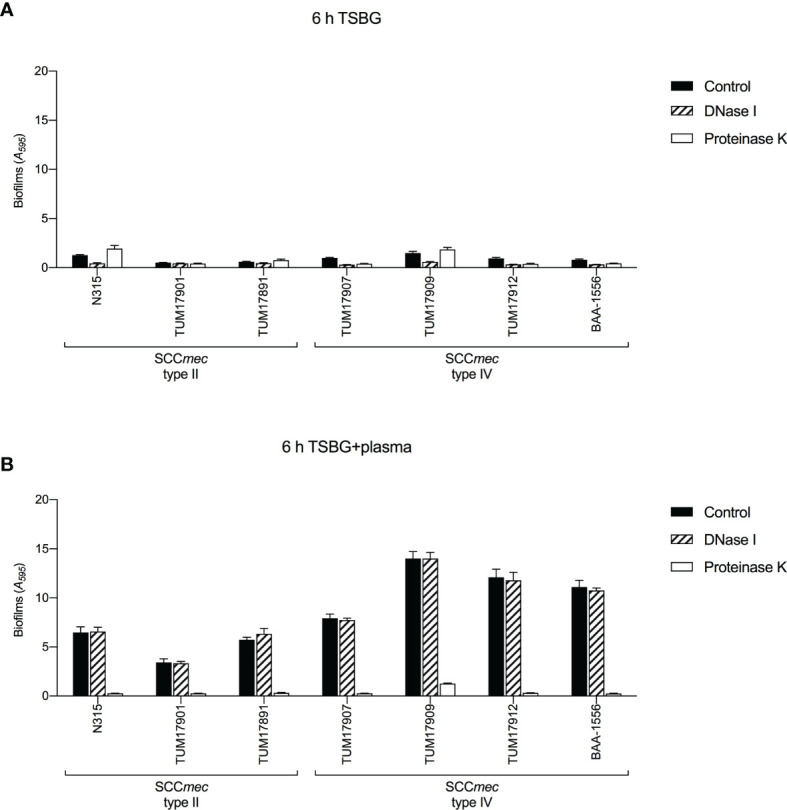
Biofilm formation tests in the presence of extracellular matrix (ECM)-degrading agents. Biofilm formation was measured by crystal violet staining assay. **(A)** The effect of DNase I and proteinase K on biofilm formation in the absence of plasma. **(B)** The effect of DNase I and proteinase K on biofilm formation in the presence of plasma. Three wells were used for each strain per experiment, and the experiment was conducted twice. Data from the total of six experiments were compared between strains. TSBG, tryptic soy broth containing 0.5% glucose; Control, biofilm without treatment.

In previous reports (Cheng et al., 2010; Zapotoczna et al., 2015; Liesenborghs et al., 2018; Trivedi et al., 2018), *S. aureus* was shown to convert the coagulation factor fibrinogen in blood plasma into fibrin and uses it as ECM in biofilms. Here, to investigate the components of ECM in biofilms of each strain with or without plasma, we used DNase I and proteinase K. In the absence of plasma, the effect of biofilm formation inhibition by DNase I and proteinase K varied among strains. DNase I inhibited biofilm formation in all SCC*mec* type IV strains (**Figure 4A**). In contrast, in the presence of plasma, the biofilm formation of all strains was inhibited by proteinase K, but not by DNase I. (**Figure 4B**). These results indicate the extensive involvement of extracellular protein components (probably the coagulation factor fibrinogen in plasma) in plasma-biofilm formation, regardless of strain type.

### Gene Transcription in Biofilm Formation

The transcript levels of coagulase (*coa*) and staphylokinase (*sak*) involved in the synthesis and degradation of fibrin and of MSCRAMMs such as fibronectin-binding protein (*fnbA*) and fibrinogen-binding protein (*fib*, *clfA*, and *clfB*) were measured by RT-qPCR. Response regulator *saeR*, sigma factor *sigB*, transcriptional regulator *sarA*, and quorum sensing system (*agrA*/*rnaIII*) were also evaluated. Transcript levels were measured after 1 h of incubation (for forming biofilm) because the plasma-biofilms formed by SCC*mec* type IV strains were already thicker than those of SCC*mec* type II strains after 2 h in the CV staining method (**Supplementary Figure 2**).

**Figure 5:**
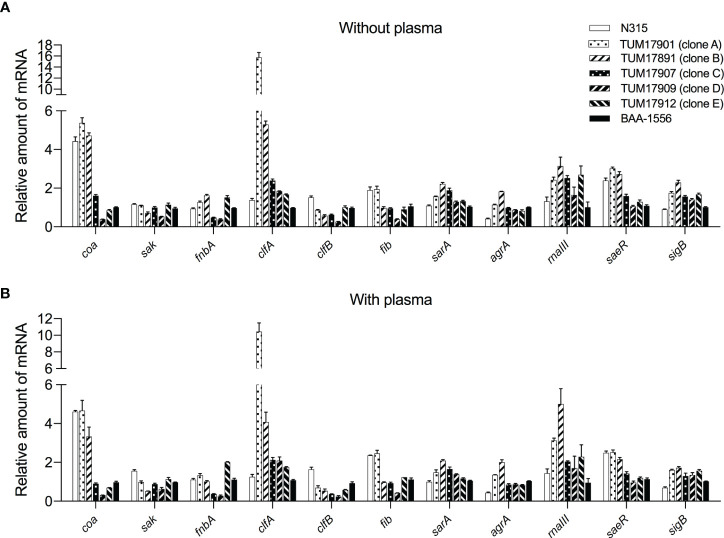
Measurement of biofilm-related gene transcription by reverse-transcription quantitative PCR (RT-qPCR). **(A)** Transcript levels of each strain after 1 h of incubation without plasma. **(B)** Transcript levels of each strain after 1 h of incubation with plasma. Two wells were used for each strain per experiment, and the experiment was conducted three times. Data from the total of three experiments were compared between strains. The data were analyzed using the ΔΔCt method. The 16S rRNA gene was used as an internal standard.

There was little difference in transcription levels among the treatments with and without plasma (**Figures 5A, B****)**. Therefore, the factors affecting plasma-biofilms could not be clarified in this experiment. However, the transcript levels of several factors varied among the strains. Transcript levels of the *coa* gene in three SCC*mec* type II strains were higher than in four SCC*mec* type IV strains. Transcript levels of the *clfA* gene in TUM17901 and TUM17891 were higher than in four SCC*mec* type IV strains and N315. Transcript levels of the *saeR* gene in three SCC*mec* type II strains were higher than in four SCC*mec* type IV strains, indicating the promotion of *coa* gene transcription *via saeR*. With plasma, transcript levels of the *agrA* and *rnaIII* genes in TUM17901 and TUM17891 were higher than in four SCC*mec* type IV strains and N315, indicating the promotion of *clfA* gene transcription *via agrA* and *rnaIII* in the presence of plasma.

## Discussion

Various types of clones have been identified as CA-MRSA in countries other than the United States, including global clones (ST30/SCC*mec* type IV) in various parts of the world, European clones (ST80/SCC*mec* type IV) in Europe, and Taiwanese clones (ST59/SCC*mec* type IV or V) in Southeast Asia. These strains share common characteristics such as classification as SCC*mec* type IV and positivity for the PVL gene. In Japan, an increasing number of CA-MRSA isolations are unique and different from those in other countries.

In this study, our data clarified that the dominant bacteremia-causing clone in our hospital changed from SCC*mec* type II (the HA-MRSA genotype) to SCC*mec* type IV (the CA-MRSA genotype) during the study period (**Figure 1**). Since most bacteremia cases develop during hospitalization, it is thought that the CA-MRSA genotype invades hospital-associated settings from community settings and spreads bloodstream infections. We investigated why SCC*mec* type IV clones are more likely to cause bloodstream infections by examining their plasma-biofilms.

We successfully characterized the relationship between the molecular characteristics of the strains and their ability to form plasma-biofilms. No significant differences in biofilm formation ability between each clone were observed in the absence of plasma; however, the plasma-biofilm formation ability of SCC*mec* type IV strains was increased compared with that of SCC*mec* type II strains in the presence of plasma, with an earlier formation of thicker structures (**Figures 2**, **3**). These thick plasma-biofilms were strongly inhibited by proteinase K, suggesting that they were robust biofilms based on coagulation proteins (**Figure 4**).

We confirmed the possession of genes that affect biofilm formation. The results showed that *fnbB*, *arcA*, and *cna* are strain-specific virulence factors, and that some of the strains do not possess these genes. In addition, the strains that possessed these genes exhibited a higher plasma-biofilm formation ability. This suggests that these strain-specific factors influence the formation of plasma-biofilm. We compared the transcript levels of the nonspecific genes possessed by all strains, because we hypothesized that differences in protein production might influence biofilm formation. However, there was no relationship between the transcriptional level of MSCRAMM, which is present in most *S. aureus* strains and the amount of plasma biofilm formation (**Figure 5**). Therefore, the presence of strain-specific factors, such as *fnbB*, *arcA*, and *cna*, has a stronger effect on formation of plasma biofilm than the expression of MSCRAMM.

In contrast to our prediction, visible floating biofilms were observed in SCC*mec* type II strains but not in SCC*mec* type IV strains, which exhibited adherent biofilms (**Supplementary Figure 3**). SCC*mec* type II clones may form floating biofilms *via* the functions of coagulase and fibrinogen-binding protein, while SCC*mec* type IV clones may adhere *via cna*, *arcA*, or *fnbB* and form mature adherent biofilms using blood plasma. It is necessary to examine the meaning of these differences in plasma-biofilm formation between SCC*mec* types.

The fact that SCC*mec* type IV clones formed robust plasma-biofilms suggests their high virulence in bacteremia. Plasma-biofilms were formed quickly and robustly; these traits are likely to facilitate the process by which MRSA enters the bloodstream, adheres to the cell wall, and proliferates in the blood vessels, which may cause high virulence in bacteremia.

Although our study was successful, this study had limitations. First, the whole genome sequences of the strains were not analyzed; there might be other factors that affect the formation of plasma-biofilms. Second, our data suggested the possibility that strain-specific genes such as *cna*, *arcA*, and *fnbB* are involved in plasma-biofilm formation. However, further analysis using gene knockout and complementation strains are necessary to prove these hypotheses. Third, the amounts of plasma-biofilm formation are clearly different between SCC*mec* type II and type IV, and the composition may also be different. However, our study suggested that the main component is protein, whether it is type II or type IV. Additional experiments, such as proteomic analysis, are required for determining the composition of the plasma biofilm of each strain, which may lead to the development of therapies specific to highly pathogenic clones, such as the SCC*mec* type IV strain.

This is the first report to demonstrate that SCC*mec* type IV strains had an increased ability to form plasma-biofilms. The results provide novel evidence regarding plasma-biofilm formation that may inform further studies of the virulence of SCC*mec* type IV strains. These findings indicate that it is necessary to prevent SCC*mec* type IV strains from invading hospital-associated settings and to design a strategy for targeting plasma-biofilm formation in bloodstream infections caused by highly virulent MRSAs.

## Data Availability Statement

The original contributions presented in the study are included in the article/**Supplementary Material**. Further inquiries can be directed to the corresponding author.

## Author Contributions

MH and TY conceived and designed this study. MS and HM isolated and stored bacterial strains from blood culture samples. MH, TY, AS, and DO performed the experiments using bacterial strains. MH and TY analyzed the data in consultation with TM, KA, CK, and SK. MH and TY wrote the paper in consultation with YI and KT. All authors contributed to the article and approved the submitted version.

## Funding

This work was supported by the JSPS KAKENHI Grant-in-Aid for Young Scientists to TY [Grant number 19K17938] and the MEXT Grant-in-Aid for Private University Research Branding Project to KT.

## Conflict of Interest

The authors declare that the research was conducted in the absence of any commercial or financial relationships that could be construed as a potential conflict of interest.

## Footnotes

1 https://janis.mhlw.go.jp/english/report/index.html

2 www.comstat.dk
