# Supplementary material for: Increased Incidence and Plasma-Biofilm Formation Ability of SCCmec Type IV Methicillin-Resistant Staphylococcus aureus (MRSA) Isolated From Patients With Bacteremia
Source: Front Cell Infect Microbiol. 2021 Mar 26;11:602833. doi: 10.3389/fcimb.2021.602833 (PMC8032974; doi:10.3389/fcimb.2021.602833)
Supplement: Supplementary file 1 [file DataSheet_1.pdf]

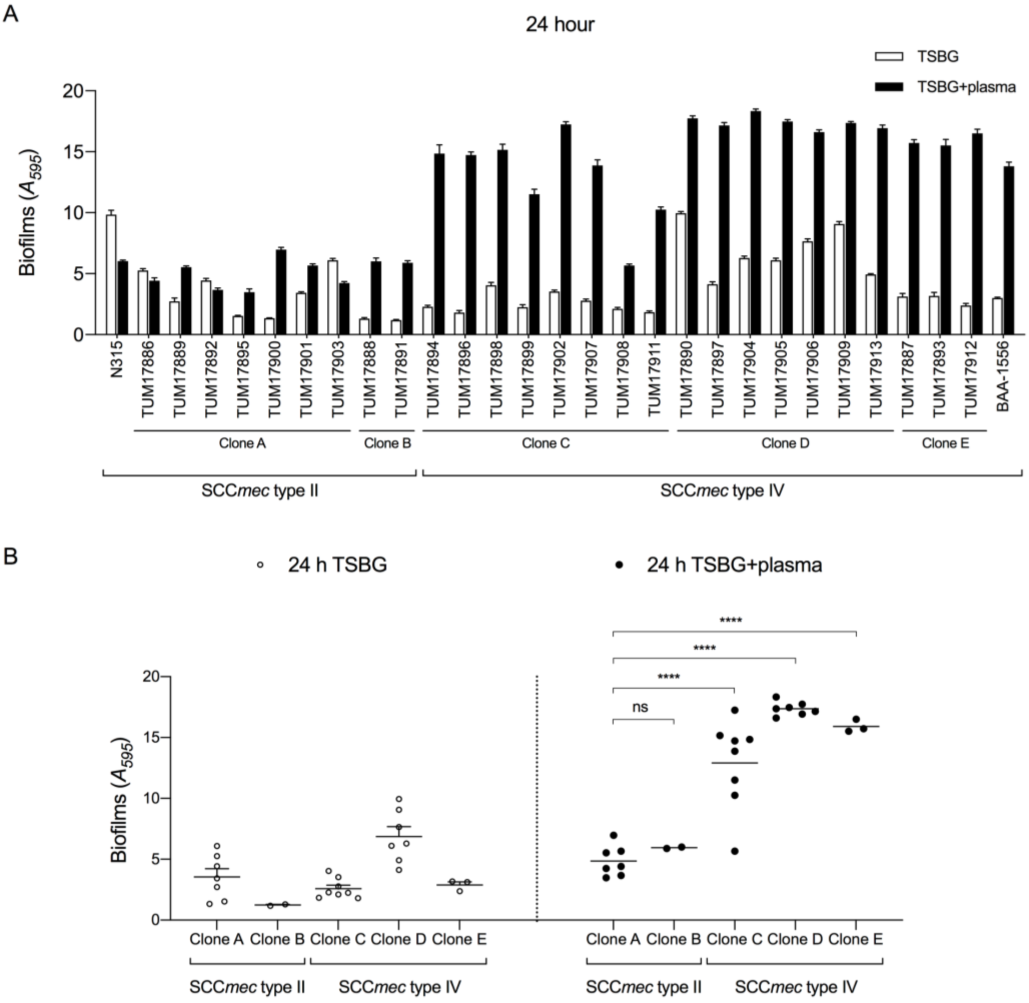

Supplementary Figure 1. Quantification of biofilms by crystal violet (CV) staining assay (24 h). (**A**) Amount of biofilm formation of each strain with or without plasma. (**B**) Comparison of biofilm formation between clones. Three wells were used for each strain per experiment, and the experiment was conducted three times. Data from the total of nine experiments were compared between strains. ****p < 0.0001; one-way ANOVA followed by Tukey's multiple comparison test was used in (**B**). TSBG, tryptic soy broth containing 0.5% glucose; ns, not significant.

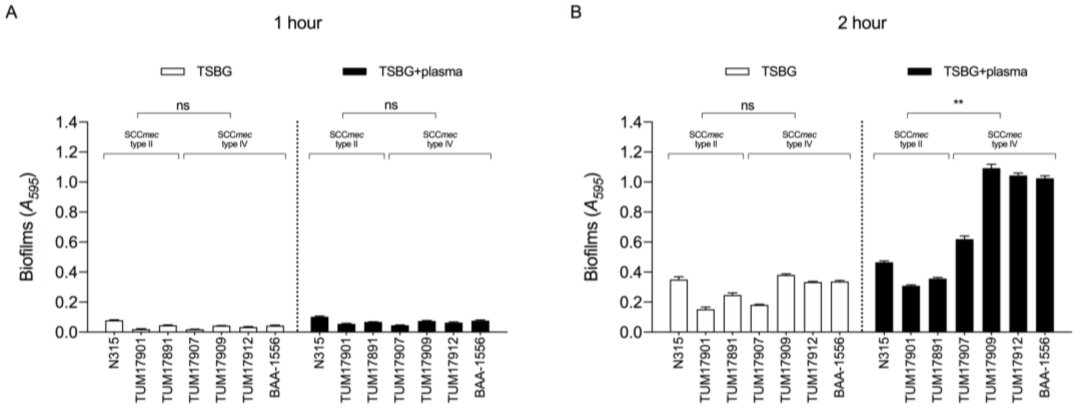

Supplementary Figure 2. Quantification of biofilms in the early phase by crystal violet (CV) staining assay. (**A**) Amount of biofilm formation of each strain after 1 h of incubation with or without plasma. (**B**) Amount of biofilm formation of each strain after 2 h of incubation with or without plasma. The results were confirmed by three independent experiments. $^{**}p < 0.005$; a *t*-test was used in (**A,B**). TSBG, tryptic soy broth containing 0.5% glucose; ns, not significant.

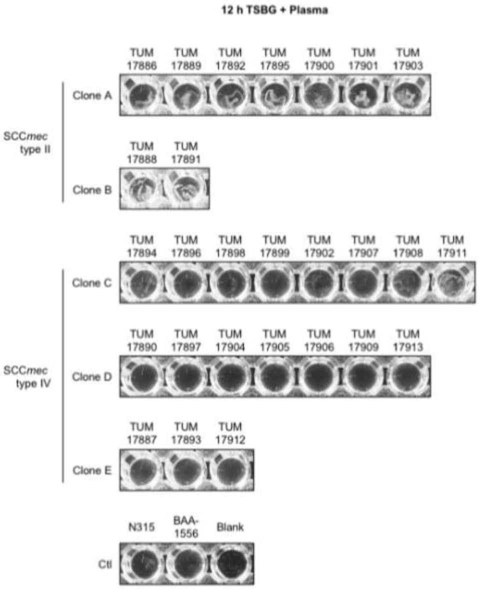

Supplementary Figure 3. Visual of floating biofilms prior to crystal violet (CV) staining assay. Before the CV staining assay, visible floating aggregates were observed in SCC*mec* type II strains, but not in SCC*mec* type IV strains, cultured in the presence of plasma. The presence of these aggregates was not confirmed in the presence of proteinase K (data not shown), indicating the involvement of extracellular protein components in aggregation. TSBG, tryptic soy broth containing 0.5% glucose.
